# Fluorescence-based cell-specific detection for laser-capture microdissection in human brain

**DOI:** 10.1038/s41598-017-14484-9

**Published:** 2017-10-27

**Authors:** Brad R. Rocco, Hyunjung Oh, Rammohan Shukla, Naguib Mechawar, Etienne Sibille

**Affiliations:** 1 Campbell Family Mental Health Research Institute, Centre for Addiction and Mental Health, Toronto, ON Canada; 2 Department of Psychiatry, University of Toronto, Toronto, ON Canada; 3 Department of Pharmacology and Toxicology, University of Toronto, Toronto, ON Canada; 4 McGill Group for Suicide Studies, Douglas Mental Health University Institute, Verdun, QC Canada; 5 Department of Psychiatry, McGill University, Montreal, QC Canada

## Abstract

Cell-specific molecular investigations of the human brain are essential for understanding the neurobiology of diseases, but are hindered by postmortem conditions and technical challenges. To address these issues we developed a multi-label fluorescence *in situ* hybridization protocol and a novel optical filter device to identify cell types and control for tissue autofluorescence. We show that these methods can be used with laser-capture microdissection for human brain tissue cell-specific molecular analysis.

## Introduction

The brain comprises numerous molecularly-distinct cell types responsible for unique functions^1^. Cell-specific alterations contribute to neurological and psychiatric disorders; however, isolating specific cell types from brain tissue of individuals afflicted with brain disorders to investigate cell-specific alterations is a challenging task. While single cells from resected human brain samples^2^ or single nuclei from fresh frozen postmortem human brain tissue^3^ have been isolated using flow cytometry techniques and subsequently classified into molecularly similar types via RNA-sequencing and post-hoc analysis, these tissue procurement and processing procedures may not be feasible for transcriptomic comparison between human subject groups. Alternatively, laser-capture microdissection (LCM) has been used to isolate cell types from human brain tissue for this purpose^4–6^. LCM requires the a priori identification of cells. In human brain tissue this has been mainly achieved using Nissl staining. This approach is limited by the few number of cell types that can be identified by the morphology and distribution of their cell body following this simple stain. By contrast, detecting cells on the basis of their molecular characteristics would allow for the identification of a much greater variety of cell types.

Recent advances in fluorescent *in situ* hybridization (FISH) have made it a reliable method for labeling mRNAs in postmortem human tissue. FISH reliably labels fresh frozen brain tissue, a common preservation method. By contrast, immunohistochemistry often requires fixative preservation, treatment with serum, which contains RNAse activity, and antigen retrieval methods, all of which can interfere with subsequent transcript analyses. FISH requires fewer processing steps than chromogenic *in situ* hybridization assays and offers a greater capacity for multi-labeling. Here, we modified a previously described FISH protocol^7^ to label molecularly distinct cell types for LCM. This method uses a series of amplification steps to increase the detectability of hybridized probes, a critical feature for detecting low-abundant transcripts. Also, the labeling conditions were optimized for shorter processing time to limit mRNA degradation for subsequent analyses.

**Figure 1:**
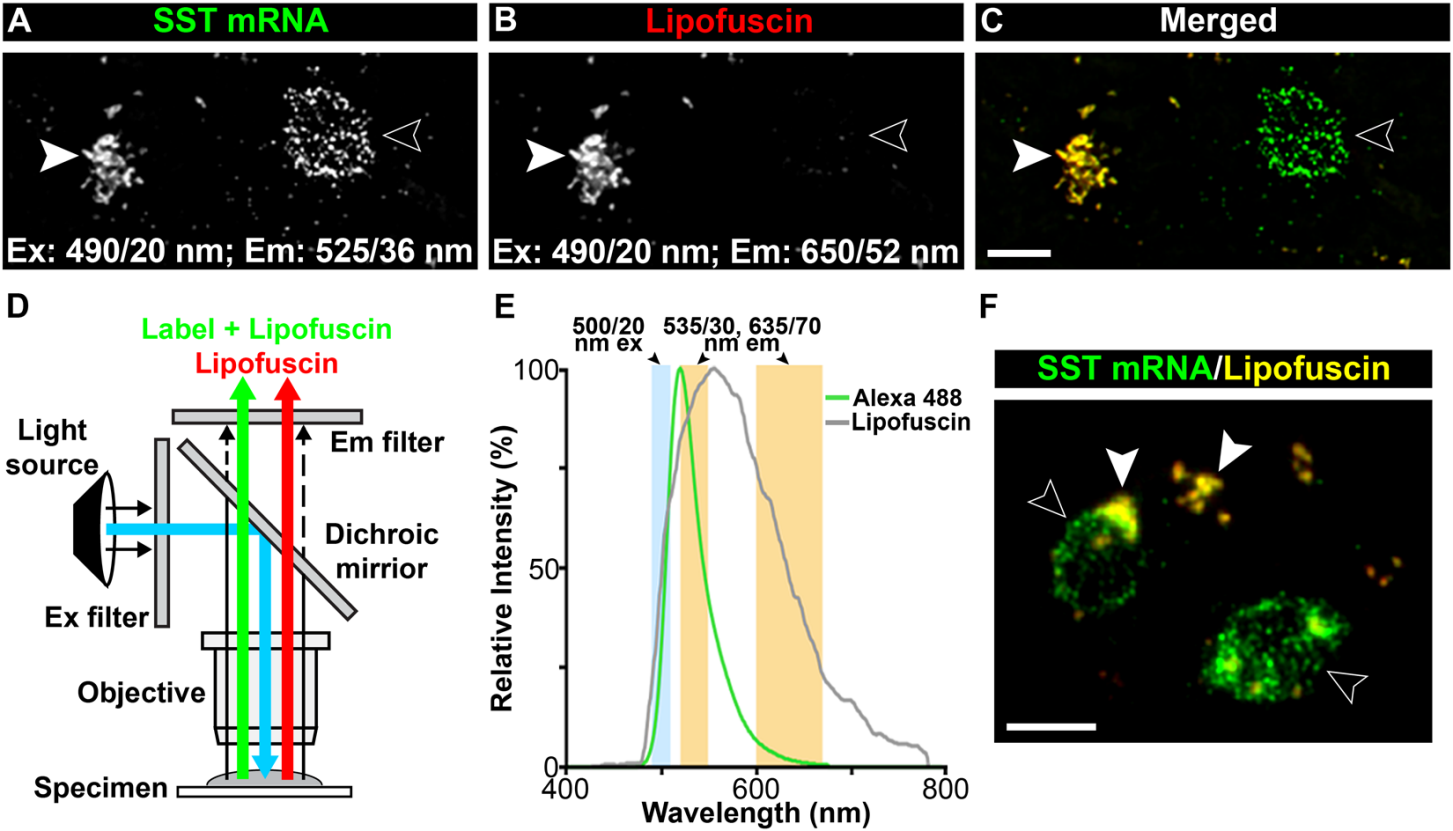
Theory and design of the custom filter cube. (**A**–**C**) Images of a human orbitofrontal cortex (OFC) tissue section labeled for somatostatin (SST) mRNA conjugated to Alexa Fluor 488. Single plane image of (**A**) SST mRNA and (**B**) lipofuscin autofluorescence. The excitation (ex) and emission (em) filter properties (wavelength/bandwidth) used for image capture are indicated. (**C**) A merged image of **A** and **B**. (**D**) Schematic illustration of the custom filter cube. (**E**) Plot showing relative em intensities for Alexa Fluor 488 and for lipofuscin autofluorescence excited by a 488 nm laser. The shaded regions correspond to the ex and em filter settings used for the custom filter cube. (**F**) Image of the same tissue section as in **A**-**C** but captured using the custom filter cube. Arrows indicate lipofuscin autofluorescence (solid arrowheads) and SST mRNA grains (open arrowheads). Bars = 10 μm.

Despite the advantages of FISH, fluorescence labeling is not widely used in human brain tissue because of inherently high levels of autofluorescence arising from lipofuscin, a by-product of lysosomal degradation that accumulates with age^8,9^. Lipofuscin has broad spectral properties^10^ that produces false positive signal across virtually all fluorescent channels (Supplementary Fig. S1) making fluorescently-labeled signals challenging to detect (Fig. 1A). Therefore, to use our FISH protocol to detect cell types in human brain tissue we had to devise a way to control for lipofuscin autofluorescence. We initially tested the application of a lipofuscin quenching reagent but found that the treatment also reduced the detectability of our fluorochrome-labeled signal (Supplementary Fig. S2), which confounds comparisons between subject groups. Therefore, we tested a novel approach that would not affect labeled signal.

Due to the broad spectral properties of lipofuscin we predicted that both fluorochrome-labeled signal and lipofuscin autofluorescence would be detected using excitation and emission filters within the required range for fluorochrome detection, but that only lipofuscin would be detected using the same excitation filter with an emission filter that is more red-shifted. We confirmed this prediction using independently-controlled excitation and emission filter components that allowed us to sequentially capture images using distinct emission filters (Fig. 1A–C). Therefore, to simultaneously detect and discriminate lipofuscin from our fluorochrome-labeled signal we custom-designed a filter cube that took advantage of these spectral properties (Fig. 1D and E). This design made Alexa Fluor 488-labeled signal easily distinguishable from lipofuscin autofluorescence (Fig. 1F) and greatly reduced autofluorescence of membrane slides that are required for LCM (Supplementary Fig. S3). Together these properties resulted in overall greater signal detectability in the presence of lipofuscin compared to a standard dual-pass filter cube (Supplementary Fig. S4).

**Figure 2:**
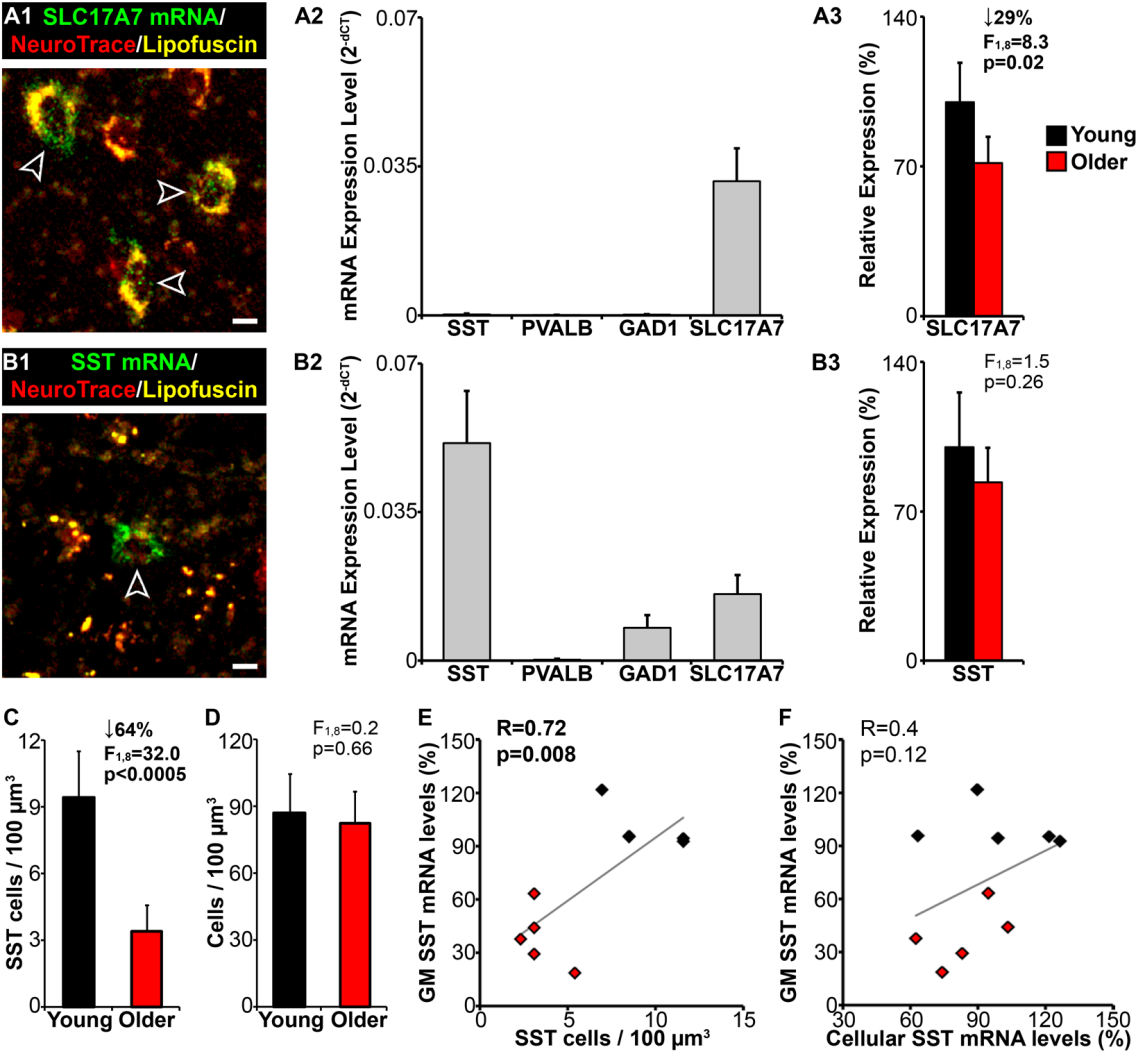
Cell-specific gene expression. (**A1** and **B1**) Images of a human OFC tissue section on a PEN membrane slide labeled for (**A1**) SLC17A7 mRNA or (**B1**) SST mRNA, counterstained with NeuroTrace red, and captured using the custom filter cube. Arrows depict SLC17A7 cells or a SST cell. Bars = 10 μm. (**A2** and **B2**) Graphs showing mean (±standard deviation [s.d.]) mRNA expression levels from LCM collected (**A2**) SLC17A7 cells or (**B2**) SST cells from all subjects. (**A3** and **B3**) Graphs showing mean (±s.d.) relative mRNA expression levels from LCM collected (**A3**) SLC17A7 cells or (**B3**) SST cells. (**C** and **D**) Mean (±s.d.) density of (**C**) SST cells and (**D**) total cells. (**E** and **F**) Plot showing correlations between relative SST mRNA levels in OFC gray matter (GM) and (**E**) SST cell density or (**F**) SST mRNA levels from LCM collected SST cells.

We next tested the utility of the FISH labeling and custom filter cube to identify distinct neuronal subtypes in human brain tissue. For this we applied the novel methods to collect SLC17A7-expressing glutamatergic cells and SST-expressing GABAergic cells via LCM from the orbitofrontal cortex (OFC) of human subjects. We then compared enrichment of gene transcript levels between these subtypes, i.e., SLC17A7 is not expressed by SST cells, SST and GAD1 are not expressed by SLC17A7 cells, and PVALB is not expressed by either cell types. As predicted, collected SLC17A7 cells expressed SLC17A7 mRNA but not SST, GAD1, nor PVALB mRNAs (Fig. 2A1 and A2), whereas collected SST cells expressed SST and GAD1 mRNAs but not PVALB mRNA (Fig. 2B1 and B2). SLC17A7 mRNA was detectable in SST cells but at levels < 50% of those found in SLC17A7 cells. Because SLC17A7 is expressed by cortical pyramidal cells, which are ~20-fold more abundant than SST cells in cortex, detectable, albeit low, SLC17A7 expression in SST cells was interpreted as background levels of SLC17A7 across the tissue thickness. These results demonstrate that our labeling and detection methods can reliably identify molecularly-distinct cell types for subsequent LCM and cell-dependent gene expression analysis, including genes targeted by the FISH probes.

To further validate these methods, we tested their usefulness for quantitatively assessing transcript differences between subject groups with known autofluorescence limitations. Specifically, we compared cell-specific transcript levels within cell types collected from young and older human subjects (Supplementary Table S1). Analyses using OFC gray matter tissue homogenates showed a trending 21% and significant 61% reduction in mRNA expression for SLC17A7 and SST, respectively, in older compared to younger subjects (Supplementary Table S2). These results may reflect reduced gene expression across cells and/or a lower number of cells expressing those genes. We therefore used our optimized FISH protocol and novel detection methods to address this question.

To assess if the lower gray matter expression represented lower mRNA expression per cell across age groups, we collected with LCM 250 SLC17A7-expressing cells and 250 SST-expressing cells in OFC samples from each subject and assessed mRNA levels with quantitative PCR. We found that SLC17A7 mRNA levels were significantly 29% lower in SLC17A7 cells of older subjects (Fig. 2A3) and correlated with gray matter SLC17A7 mRNA expression (R = 0.88; p < 0.0005; Supplementary Fig. S5), suggesting a reduction in SLC17A7 expression per cell with aging. By contrast, despite considerably lower gray matter SST expression in the older subjects, SST mRNA levels in SST cells were only nominally and non-significantly decreased in older subjects (Fig. 2B3). To test the alternative hypothesis (i.e., fewer SST-expressing cells with age) we used the same FISH protocol and quantitative fluorescence microscopy methods to assess the amount of SST mRNA grains in SST cells between groups (Supplementary Fig. S6). The number of SST mRNA grains per SST cell was only trending lower in the older subjects, although the tissue grain density was significantly reduced by 49% in the older group. These findings were not biased by the amount of lipofuscin, which was significantly > 2-fold greater at the tissue level, but not in SST cells, in older subjects (Supplementary Fig. S7), as the amount of lipofuscin did not correlate with SST mRNA tissue grain density for young (R = −0.55; p = 0.15) or older (R = 0.44; p = 0.22) subjects or SST mRNA grains per SST cell for either group (young: R = 0.51; p = 0.18; older: R = 0.48; p = 0.2). Because these results are similar to our quantitative PCR finding of only a modest change in SST mRNA expression in the LCM collected SST cells, our cell-specific mRNA grain counting approach was a viable quantitative strategy to assess the amount of SST mRNA grains per SST cell.

Consistent with the grain counting data, we used a semi-automated unbiased stereological approach to assess cell density and found that the density of SST cells was 64% lower in older subjects (Fig. 2C). This finding was not due to a difference in total detectable cells between groups (Fig. 2D), and was replicated using a manual approach to identify SST cells for density assessment (Supplementary Fig. S8). Gray matter SST mRNA levels were significantly correlated with SST cell density (Fig. 2E), but not with cellular SST mRNA levels (Fig. 2F). Thus, reduced SST expression with aging is principally due to a marked reduction of SST expression in ~60% of SST cells rather than reduced expression per cell. Lower SST expression is implicated in many brain disorders^11,12^. Future studies will assess if lower SST expression in brain disorders affects a proportion of SST cells, similar to aging, or lower expression across cells.

In concert, our results demonstrate that our FISH protocol and custom filter cube can be used to overcome challenges of detecting unique cell types for LCM and subsequent cell-specific analyses. While other protocols may prove to be sufficient for labeling, the general design of the custom filter cube was crucial for simultaneously detecting multiple fluorescent labels and autofluorescence. Our results also highlight the importance of doing parallel histological studies when investigating the extent of cell-specific alterations between subject groups. These methods thus lay the groundwork for assessing cell-specific transcriptomic alterations between human subject groups.

## Methods

### Human brain samples

Tissue blocks (1–3 cm thick) containing the orbitofrontal cortex (OFC) from 5 young and 5 older male human subjects (Supplementary Table S1) with no known psychiatric or neurological disorder were provided by the Douglas-Bell Canada Brain Bank (Douglas Mental Health University Institute, Montreal, QC, Canada). The subjects were selected based on age and prior analysis of SST mRNA expression levels in gray matter homogenates of the OFC^13^, and matched as closely as possible for postmortem interval (PMI), RNA integrity number (RIN), and pH.

The tissue blocks were sectioned via cryostat using a 14 µm block advance. Tissue sections were mounted onto RNAse AWAY® (ThermoFisher Scientific) treated polyethylene naphthalate (PEN) membrane slides (Leica Microsystems Inc., Concord, ON, Canada), which were used for laser-capture microdissection (LCM), and every fifth section was mounted onto a glass slide and used for wide-field fluorescence microscopy. All slides were stored at −80 °C until processed for fluorescent *in situ* hybridization (FISH).

### Fluorescence ***in situ*** hybridization

The *in situ* hybridization probes used to label mRNA encoding somatostatin (SST; product # 310591) or vesicular glutamate transporter 1 (SLC17A7; product # 415611) were designed by Advanced Cell Diagnostics (Newark, CA, USA). The probe diluent (product # 300041), protease (Protease IV; product # 322340), fluorescent multiplex detection (AMP 1-4; product # 320851), and wash buffer (product # 310091) reagents, and the HybEZ Hybridization System (product # 321461) were also from Advanced Cell Diagnostics. Separate experiments were performed to label tissue sections for SST mRNA or SLC17A7 mRNA for LCM. Two experiments were performed, each using one section from all subjects, to label sections for SST mRNA for quantitative fluorescence microscopy analyses. A modified version of the RNAscope® Assay^7^ was used to label all sections as follows. H_2_O was treated with 0.1% diethyl pyrocarbonate (DEPC; Bio Basic Inc., Markham, ON, Canada), and the 1X phosphate buffered saline (PBS) and 1X wash buffer (WB) solutions were each diluted in the DEPC-treated H_2_O. Slides were taken from −80 °C and immediately immersed in chilled 4% paraformaldehyde diluted in PBS for 5 minutes. Next, the slides were serially dipped in 50% and 70% EtOH diluted in H_2_O, and twice in 100% EtOH (all at room temperature [RT]), and then completely dried at RT. A hydrophobic barrier pen was then used to make a barrier around the perimeter of each section and the barrier was completely dried at RT. The sections were then treated with Protease IV for 15 minutes at RT, rinsed twice in PBS at RT, and then incubated with probe solution diluted to 1:50 with probe diluent for 1 hour at 40 °C using the HybEZ Hybridization System. Next, the sections were rinsed twice in WB at RT and then successively treated with AMP 1 (15 minutes), AMP 2 (7 minutes), AMP 3 (15 minutes), and AMP 4 (7 minutes) reagents at 40 °C using the HybEZ Hybridization System. After each AMP treatment the sections were rinsed twice in WB at RT. The amplification process resulted in the conjugation of Alexa 488 to the target mRNA. After the last WB rinse the sections were rinsed twice in PBS at RT, treated with NeuroTrace red fluorescent Nissl stain (product # N21482; Molecular Probes) diluted to 1:300 in PBS for 10 minutes at RT, and rinsed twice in PBS at RT. Sections on PEN membrane slides were next dipped in 100% EtOH, completely dried at RT, and then immediately prepared for LCM. Sections on glass slides were counterstained with DAPI, sealed with a coverslip using ProLong Gold Antifade reagent (product # P36930; Invitrogen), and then stored at 4 °C until imaged. All slides were coded to conceal subject information. The entire labeling procedure took only ~2.5 hours to complete.

### Laser-capture microdissection

SST mRNA-expressing or SLC17A7 mRNA-expressing cells within the gray matter of the OFC were collected using a 20 × 0.4 NA objective on a Leica LMD7 laser microdissection microscope equipped with a Leica LMD CC7000 digital color CCD camera and a novel custom-designed filter cube. Components for the filter cube were obtained from Chroma Technology Corp. (Bellow Falls, VT, USA) and consisted of a single-pass excitation (ex) filter (500 nm [20 nm bandwidth] wavelength; product # ET500/20x), a multi-pass emission (em) filter (535 nm [30 nm bandwidth] and 635 nm [70 nm bandwidth] wavelength; product # 59026 m), and a dichroic mirror (product # 69008bs). The spectral properties of the novel filter cube (Fig. 1E) were specifically designed to simultaneously detect and distinguish Alexa Fluor 488-labeled signal, NeuroTrace red-labeled signal, and lipofuscin autofluorescence (Fig. 2A1 and B1), and to reduce inherent autofluorescence of membrane slides (Supplementary Fig. S3).

250 NeuroTrace labeled cells that expressed SST mRNA or SLC17A7 mRNA per subject were captured via LCM. The cells were collected into nuclease-free 0.6 mL microcentrifuge tubes containing 50 µL of extraction buffer provided in the PicoPure® RNA Isolation Kit (Applied Biosystems), incubated at 42 °C for 30 minutes, and then stored at −80 °C until processed for RNA extraction and purification.

### RNA extraction

The PicoPure RNA Isolation Kit and standard protocol was used for RNA extraction and purification. Briefly, the extraction buffer solution containing the dissected cells was removed from −80 °C and thawed at RT. 50 µL of 70% EtOH was added to the solution and then the entire volume was added to the purification column and rinsed with wash buffer. 40 µL of DNAse treatment (RNAse-free DNAse set; Qiagen) was added to the purification column for 15 minutes at RT, rinsed with a series of wash buffers, and then the purified RNA was eluted with 15 µL of elution buffer. This kit and protocol were also used to extract and purify RNA from the homogenized OFC gray matter samples.

### cDNA synthesis and quantitative PCR

cDNA was synthesized using the SuperScript® VILO cDNA synthesis kit (Invitrogen) and stored at −20 °C until processed for quantitative PCR. PCR products were amplified in triplicate with a CFX96 real-time system (Bio-Rad Laboratories, Mississauga, ON, Canada) using universal PCR conditions. Results were calculated as the geometric mean of threshold cycles normalized to genes encoding actin beta (ACTB) and glyceraldehyde-3-phosphate dehydrogenase (GAPDH). The genes tested include those encoding somatostatin (SST), vesicular glutamate transporter 1 (SLC17A7), glutamic acid decarboxylase 67 (GAD1), and parvalbumin (PVALB). This kit and protocol were also used to quantify ACTB, GAPDH, SST, and SLC17A7 from the homogenized OFC gray matter samples.

### Wide-field fluorescence microscopy

Data were collected on an Olympus IX83 microscope (Richmond Hill, ON, Canada) equipped with a Hamamatsu ORCA-Flash4.0 V2 digital CMOS camera (Bridgewater, NJ, USA) and high-precision ProScan III XYZ motorized stage with linear XYZ encoders (Prior Scientific, Rockland, MA, USA). One experiment was collected using a 20 × 0.75 NA objective and the other was collected using a 60 × 0.40 NA super corrected oil immersion objective. The hardware was controlled by SlideBook 6 (Intelligent Imaging Innovations, Inc., Denver, CO, USA), which was the same software used for post-acquisition processing. For each experiment, 20 randomly sampled 3-D image stacks (2-D images sequentially captured at intervals separated by 0.75 μm and 0.25 μm in the z-dimension for the 20× and 60× objectives, respectively) were acquired within the gray matter of the OFC at the approximate location of the LCM collected SST cells using a sampling grid of 500 × 500 μm. The stacks were 1024 × 1024 pixels (~333 × 333 μm and ~111 × 111 μm for the 20× and 60× objectives, respectively) and were acquired over the entire thickness of the tissue section using optimal exposure settings. Differences in exposures were normalized during image processing. The Sedat Quad 89000 filter set (Chroma Technology Corp.) was used to detect each fluorescent channel. The ex and em wavelengths used to detect DAPI, SST mRNA, and NeuroTrace red were 402 nm (15 nm bandwidth) ex/455 nm (50 nm bandwidth) em, 490 nm (20 nm bandwidth) ex/525 nm (36 nm bandwidth) em, and 555 nm (25 nm bandwidth) ex/605 nm (52 nm bandwidth) em, respectively. Lipofuscin autofluorescence was detected using 402 nm (15 nm bandwidth) ex/705 nm (72 nm bandwidth) em wavelengths.

### Image processing

Each fluorescent channel was deconvolved using the AutoQuant adaptive blind deconvolution algorithm (Media Cybernetics, Inc., Rockville, MD, USA). Data segmentation was performed on the channels used to detect NeuroTrace, SST mRNA, and lipofuscin from the stacks collected using the 60x objective. A flow chart illustrating the entire process is provided in Supplementary Fig. S9. NeuroTrace labeled cells were manually segmented only if they 1) contained a DAPI labeled nucleus, 2) were continuous across several z-planes, 3) had a clearly distinguishable center, defined as the z-plane containing the largest surface area of the cell, that was not located on the first or last z-plane of the stack, and 4) were contained within an unbiased counting frame (~68 × 68 μm) placed in the center of each stack and consisting of 2 exclusion lines and 2 inclusion lines. The perimeter of the center of each cell meeting these criteria was manually traced and filled, and then the resulting mask was copied to the adjacent top and bottom z-plane.

Segmentation of SST mRNA grains and lipofuscin was done in MATLAB (R2016). Initially, a new channel that was used for data segmentation only was created from each deconvolved channel by calculating a difference of 3-D Gaussian filtered channels using sigma values of 0.7 and 2. Lipofuscin was segmented using the Ridler-Calvard automated thresholding algorithm^14^. SST mRNA grains were segmented using an iterative segmentation algorithm as previously described^15^ but with a few exceptions. Specifically, the Ridler-Calvard automated thresholding algorithm was used to obtain an initial iterative segmentation value with successive iterations increasing by 25 values. After each iteration, the segmented object masks were sized-gated between 0.03–0.1 μm^3^ and the iterations were performed until the last segmentation value produced all object masks ≤ 0.1 μm^3^. All SST mRNA object masks that overlapped a lipofuscin mask were excluded from analysis to control for potential false positive signal resulting from lipofuscin autofluorescence (Supplementary Fig. S6c–e). SST mRNA object masks that overlapped a cell mask were assigned to that cell. After segmentation each image was manually inspected for masking accuracy.

### Classification of SST cells

For analysis of the stacks collected using the 60x objective, cells containing ≥ 20 SST mRNA grains were considered to be SST cells. This threshold was based on prior findings using a similar approach for defining cell types by mRNA expression^15^ and the distribution of SST mRNA grains per cell (Supplementary Fig. S6f).

For analysis of the stacks collected using the 20x objective, all NeuroTrace labeled cells containing a DAPI labeled nucleus that were located within an unbiased counting frame (~250 × 250 μm) placed in the center of each stack and consisting of 2 inclusion and 2 exclusion lines were included for analysis. Compared to the stacks collected using the 60× objective, SST mRNA grains resembled more cluster-like structures in the stacks collected using the 20× objective. Therefore, SST cells were manually identified by the presence of ≥10 SST mRNA grain clusters within the NeuroTrace/DAPI labeled cell (Supplementary Fig. S8a), which presumably represented a greater number of SST mRNA grains per cell at this resolution.

### TrueBlack experiment: labeling, imaging, and data segmentation

Two adjacent prefrontal cortex tissue sections (12 μm thick) from 5 subjects with no known neurological disorder were used. The sections were mounted on glass slides and the protocol under *Fluorescent in situ hybridization* was used to label each section for mRNAs encoding SLC17A7 and the neuron enriched gene encoding enolase 2 (ENO2; designed by Advanced Cell Diagnostics), which were conjugated to Alexa 488 and Atto 550, respectively. After the last WB rinse the sections were rinsed in PBS, and 1 section from each subject was counterstained with DAPI and sealed with a coverslip using ProLong Gold Antifade reagent. The other section was treated with TrueBlack Lipofuscin Autofluorescence Quencher (Biotium Inc. Fremont, CA, USA) according to the manufacturer’s protocol. Briefly, TrueBlack was diluted to 1X using 70% EtOH, the sections were incubated with TrueBlack for 30 seconds, rinsed in PBS, counterstained with DAPI, and sealed with a coverslip using ProLong Gold Antifade reagent. All sections were stored at 4 °C until imaged.

The sections were imaged with the same hardware and software stated under *Wide-field fluorescence microscopy* using a 60 × 1.40 N.A super corrected oil immersion objective. Ten randomly sampled 3-D image stacks (2-D images successively captured at intervals separated by 0.25 μm in the z-dimension) that were 1024 × 1024 pixels (~111 × 111 μm) were acquired over the entire thickness of the tissue section using a sampling grid of 500 × 500 μm. The same area was sampled for each section per subject. The ex and em wavelengths used to detect DAPI, SLC17A7 mRNA, and ENO2 mRNA were 402 nm ex/455 nm em, 490 nm ex/525 nm em, and 555 nm ex/605 nm em, respectively. Lipofuscin autofluorescence was detected using 402 nm ex/705 nm em wavelengths.

The channels were deconvolved using the AutoQuant adaptive blind deconvolution algorithm. For data segmentation of the lipofuscin autofluorescence and DAPI channels a single normalized threshold setting for each channel was used. An iterative segmentation algorithm was used to segment the SLC17A7 mRNA grains and ENO2 mRNA grains as described in *Image Processing*. However, a single normalized threshold setting was used for the initial segmentation value. Using a single normalized threshold setting controlled for robust differences in signal intensity and detectability, which we predicted would occur across all channels as a result of the TrueBlack treatment. All SLC17A7 mRNA and ENO2 mRNA object masks that overlapped lipofuscin autofluorescence were excluded from analysis.

### Spectral Imaging

The transmission percentage of lipofuscin autofluorescence was quantified using an Olympus FV1200 laser scanning confocal microscope with Olympus FluoView software (Fig. 1E and Supplementary Fig. S1b and c). The spectral properties of Alexa 488 were obtained from ThermoFisher Scientific’s Fluorescent SpectraViewer (www.thermofisher.com/ca/en/home/life-science/cell-analysis/labeling-chemistry/fluorescence-spectraviewer.html; Fig. 1E). The spectral properties of the filters were obtained from Chroma’s Spectra Viewer (www.chroma.com/spectra-viewer; Fig. 1E).

### Statistics

An independent samples t-test was used to assess differences in mean age, PMI, RIN, and pH. An analysis of covariance model that included PMI, RIN, and pH as covariates was used to analyze all other data between subject groups; reported statistics only include covariates that were statistically significant. A paired samples t-test was used to assess data collected from the TrueBlack experiment. All statistical tests were conducted as two-tailed with α-level = 0.05, and all results are reported as mean ± standard deviation.

## Electronic supplementary material

Supplementary Information
