# Supplementary material for: Fluorescence-based cell-specific detection for laser-capture microdissection in human brain
Source: Sci Rep. 2017 Oct 27;7:14213. doi: 10.1038/s41598-017-14484-9 (PMC5660154; doi:10.1038/s41598-017-14484-9)
Supplement: Supplementary file 1 — Supplementary Information [file 41598_2017_14484_MOESM1_ESM.doc]

**Supplementary Information for**

**FLUORESCENCE-BASED CELL-SPECIFIC DETECTION FOR LASER-CAPTURE MICRODISSECTION IN HUMAN BRAIN**

Brad R. Rocco, Hyunjung Oh, Rammohan Shukla, Naguib Mechawar, & Etienne Sibille

1. Supplementary Figure S1

2. Supplementary Figure S2

3. Supplementary Figure S3

4. Supplementary Figure S4

5. Supplementary Figure S5

6. Supplementary Figure S6

7. Supplementary Figure S7

8. Supplementary Figure S8

9. Supplementary Figure S9

10. Supplementary Table S1

11. Supplementary Table S2

**SUPPLEMENTARY FIGURE LEGENDS**

**Supplementary Figure S1. Lipofuscin has inherently broad excitation and emission spectral properties.** (a) Images of an unlabeled human orbitofrontal cortex (OFC) tissue section showing lipofuscin autofluorescence that was captured using (a1) 402 nm excitation (ex; 15 nm bandwidth) and 455 nm emission (em; 50 nm bandwidth), (a2) 490 nm ex (20 nm bandwidth) and 525 nm em (36 nm bandwidth), (a3) 555 nm ex (25 nm bandwidth) and 605 nm em (52 nm bandwidth), (a4) 645 nm ex (30 nm bandwidth) and 705 nm em (72 nm bandwidth) filters. Bar = 10 μm. (b and c) Relative intensity of lipofuscin autofluorescence across wavelengths for (b) individual ex lasers or (c) across all ex lasers indicated in b.

**Supplementary Figure S2. TrueBlack lipofuscin autofluorescence quenching reagent reduces the detectability of lipofuscin autofluorescence as well as signal from fluorescence-labeling applied before and after TrueBlack treatment.** (a1-d1 and a2-d2) Images of lipofuscin autofluorescence, SLC17A7 mRNA, ENO2 mRNA, and DAPI in a prefrontal cortex tissue section from the same human subject without (a1-d1) or with (a2-d2) TrueBlack (Biotium Inc.) treatment. SLC17A7 mRNA and ENO2 mRNA was labeled with Alexa Fluor 488 and Atto 550, respectively, prior to TrueBlack treatment and DAPI labeling was applied after treatment according to the manufacturer’s protocol. Bar = 20 μm. (a3-d3) Plots of mean intensity levels of (a3) lipofuscin, (b3) SLC17A7 mRNA, (c3) ENO2 mRNA, and (d3) DAPI from sections without and with TrueBlack treatment. (a4-d4) Plots showing total detectable fluorescence signal as indicated by total volume or mRNA grain density for (a4) lipofuscin, (b4) SLC17A7 mRNA, (c4) ENO2 mRNA, and (d4) DAPI in sections without or with TrueBlack treatment. Data points of the same color depict sections without or with TrueBlack treatment from the same subject. Bolded statistics indicate a statistically significant difference, though the other statistics are trending towards statistical significance.

**Supplementary Figure S3. Membrane slide autofluorescence is greatly reduced with the custom filter cube.** (a1-c1) Images of the same region showing autofluorescence of PEN membrane slides captured using the (a1) custom filter cube, (b1) a GFP/RFP filter cube (470 nm [40 nm bandwidth]/550 nm [20 nm bandwidth] excitation wavelengths and 520 nm [30 nm bandwidth]/590 nm [40 nm bandwidth] emission wavelengths), or (c1) a GFP filter cube (470 nm [40 nm bandwidth] excitation wavelength and 525 nm [50 nm bandwidth] emission wavelength). (a2-c2) A threshold segmentation mask (red) was generated from c1 and overlaid on the images from (a2) a1, (b2) b1, and (c2) c1 to obtain fluorescence intensity levels. Intensity levels of membrane slide autofluorescence were 96% and 92% lower for the custom filter cube (18 arbitrary units [a. u.]) compared to the GFP filter cube (510 a. u.) and GFP/RFP filter cube (222 a. u.), respectively. Bar = 10 μm.

**Supplementary Figure S4. The detectability of fluorochrome-labeled signal is enhanced with the custom filter cube.** (a and a’) Images of the same region from a human OFC tissue section labeled for SLC17A7 mRNA and captured using the (a) custom filter cube or (a’) a GFP/RFP filter cube (470 nm [40 nm bandwidth]/550 nm [20 nm bandwidth] excitation wavelengths and 520 nm [30 nm bandwidth]/590 nm [40 nm bandwidth] emission wavelengths). Bar = 10 μm. (b1-b3 and b1’-b3’) Plots showing pixel intensity levels (arbitrary units [a. u.]) of SLC17A7 mRNA (green) and lipofuscin (red) across the lines in (b1-b3) a or (b1’-b3’) a’. Overlapping intensity levels are shown in yellow.

**Supplementary Figure S5. Cellular SLC17A7 mRNA levels significantly correlate with gray matter SLC17A7 mRNA levels.** Plot showing the correlation between relative SLC17A7 mRNA levels from LCM collected SLC17A7 cells and relative SLC17A7 mRNA levels from OFC gray matter (GM) homogenates from young and older subjects.

**Supplementary Figure S6. Quantification of SST mRNA grains per cell.** (a-e) Projection image (3 z-planes separated by 0.25 μm) of a human OFC tissue section labeled for SST mRNA and counterstained with NeuroTrace red and DAPI. (a and b) Images showing SST mRNA, DAPI, and (a) NeuroTrace red, or (b) Lipofuscin. (c and d) Single channel images of the boxed region in b showing (c) SST mRNA and (d) lipofuscin overlaid with their corresponding object masks. (e) The same image as c showing SST object masks that were classified as SST mRNA grains (green) and SST object masks that were classified as lipofuscin (red). Bar = 10 μm. (f) Histograms (bin width = 10) of SST mRNA grains in each cell analyzed for the young and older subject group. The arrows indicate the bin with cells containing 20-29 SST mRNA grains. Cells containing ≥ 20 SST mRNA grains were defined as SST cells. (g and h) Bar graphs showing (g) the mean (± s.d.) number of SST mRNA grains per SST cell and (h) the mean (± s.d.) density of all SST mRNA grains for the young and older subjects.

**Supplementary Figure S7. SST mRNA grain density was not affected by the amount of lipofuscin.** (a) Projection images (3 z-planes separated by 0.25 μm) of a human OFC tissue section from a young and older subject showing lipofuscin autofluorescence. Bar = 10 μm. (b) Bar graph showing the mean (± s.d.) total lipofuscin volume, a measure of the total amount of lipofuscin detected, for young and older subjects. (c) Plot showing correlations between total lipofuscin volume and the density of all SST mRNA grains for the young and older subjects. (d) Bar graph showing the mean (± s.d.) lipofuscin volume in SST cells for the young and older subjects. (e) Plot showing correlations between lipofuscin volume in SST cells and the number of SST mRNA grains per SST cell for the young and older subjects.

**Supplementary Figure S8. The density of SST cells is lower in older subjects.** (a) Projection images (3 z-planes separated by 0.25 μm) of a human OFC tissue section from a young and older subject labeled for SST mRNA and counterstained for NeuroTrace red and DAPI. Arrows indicate SST cells. Cells were manually identified as SST cells and included for analysis if they contained ≥ 10 SST mRNA grain clusters. Bar = 10 μm. (b) Bar graph showing the mean (± s.d.) density of SST cells for the young and older subjects.

**Supplementary Figure S9. Flow chart illustrating the image processing workflow.** The flow chart corresponds to the *Image Processing* section under Methods.

**SUPPLEMENTARY FIGURES**

**Supplementary Figure S1.**

**
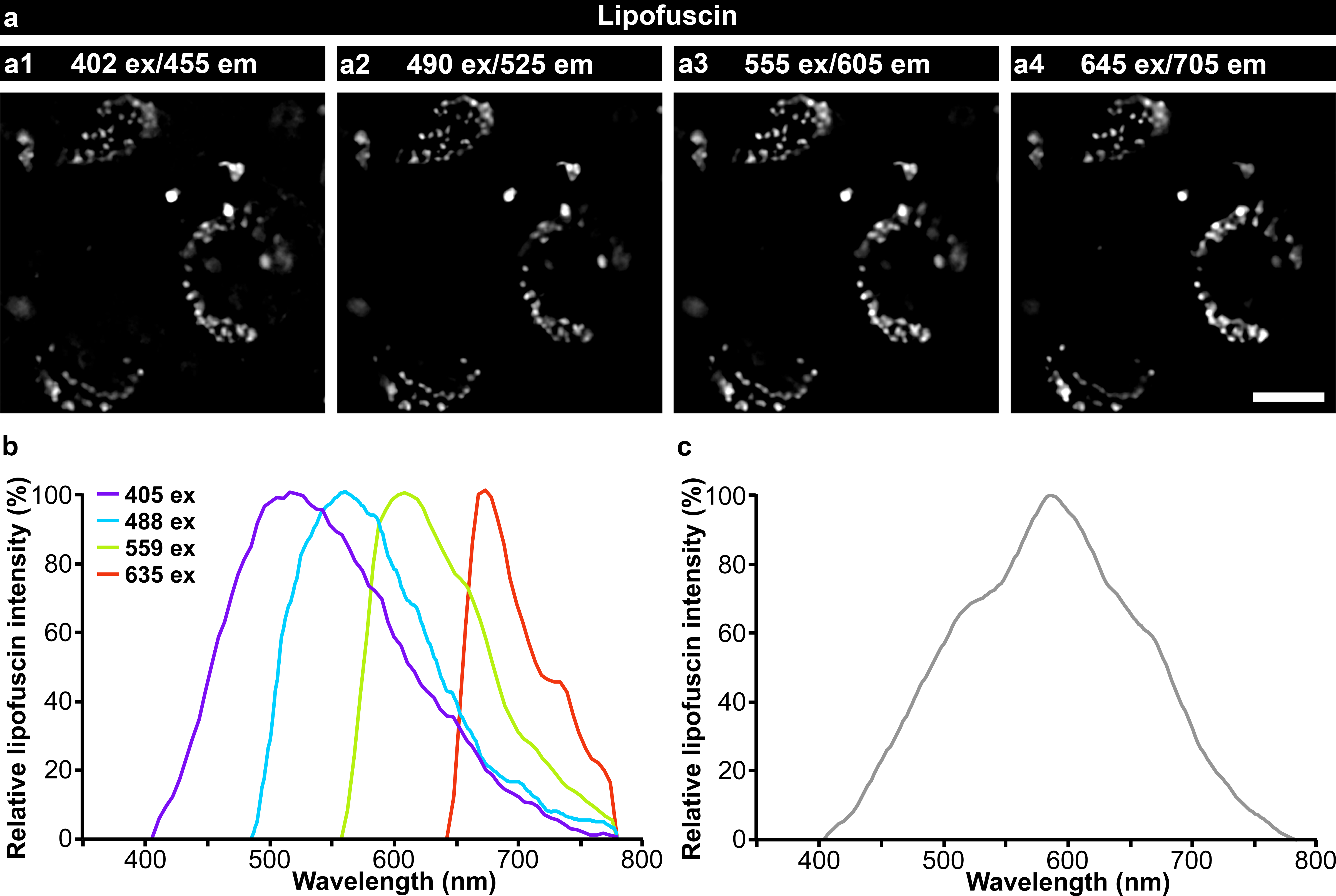
**

**Supplementary Figure S2.**

**
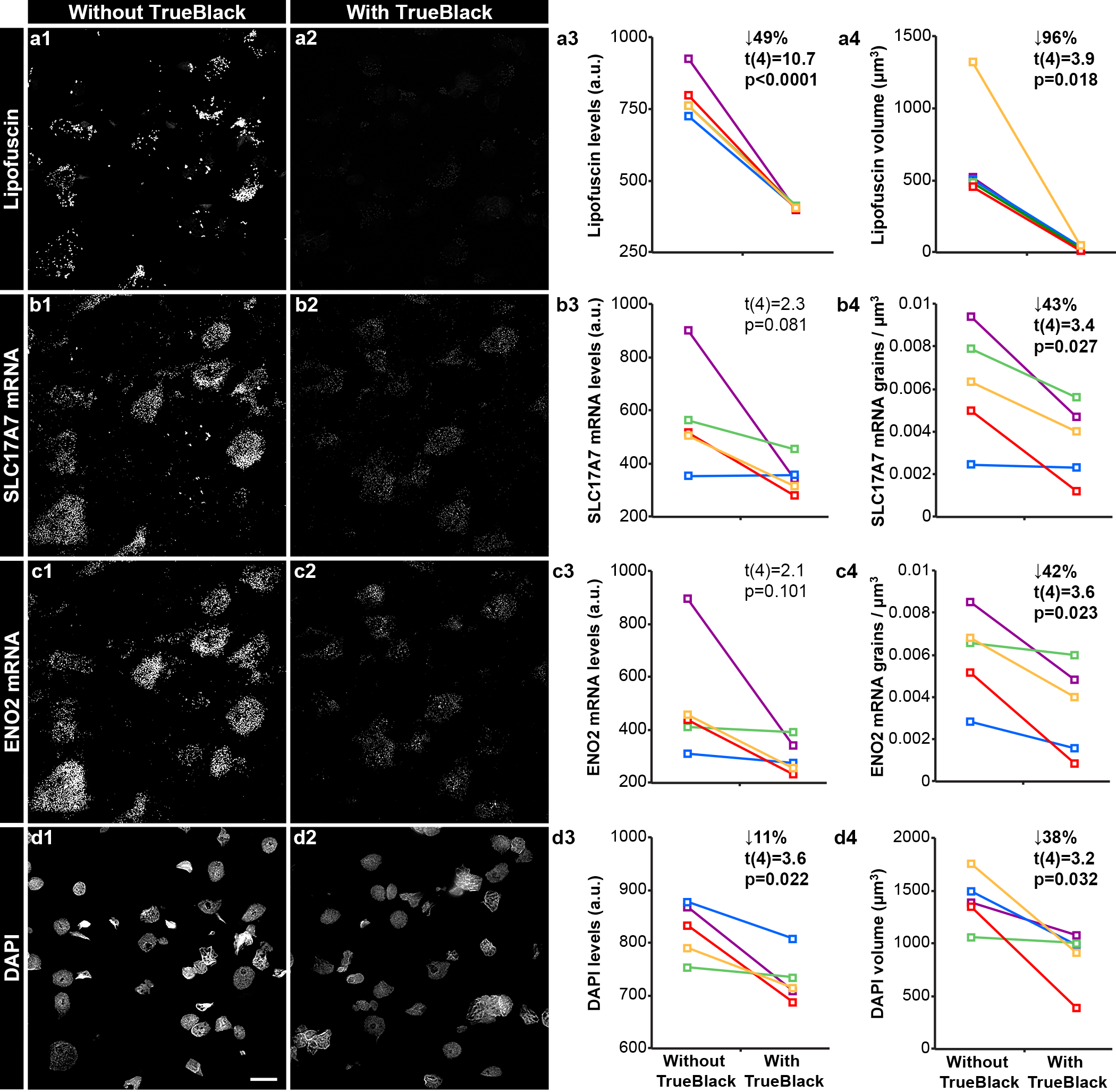
**

**Supplementary Figure S3.**

**
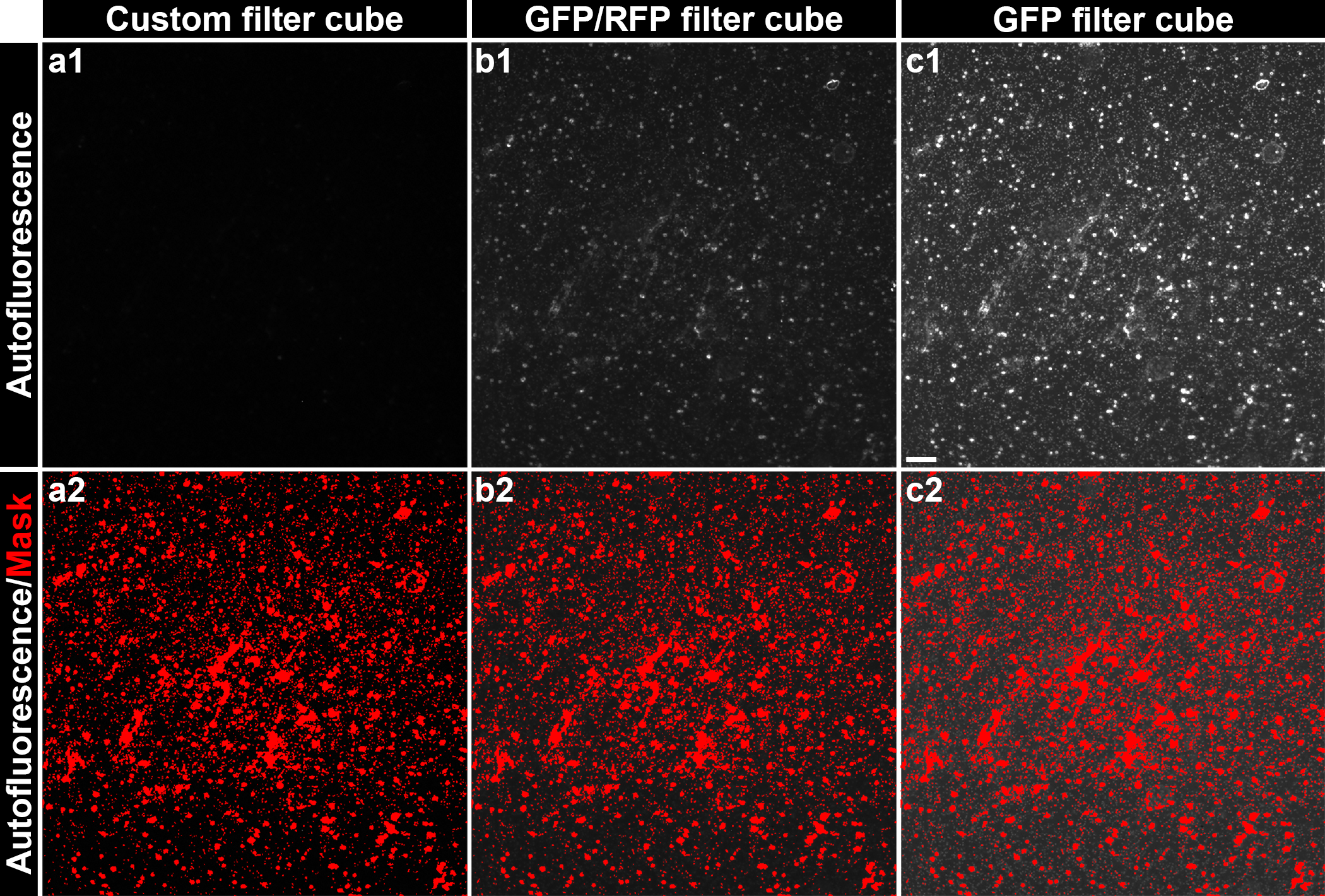
**

**Supplementary Figure S4.**

**
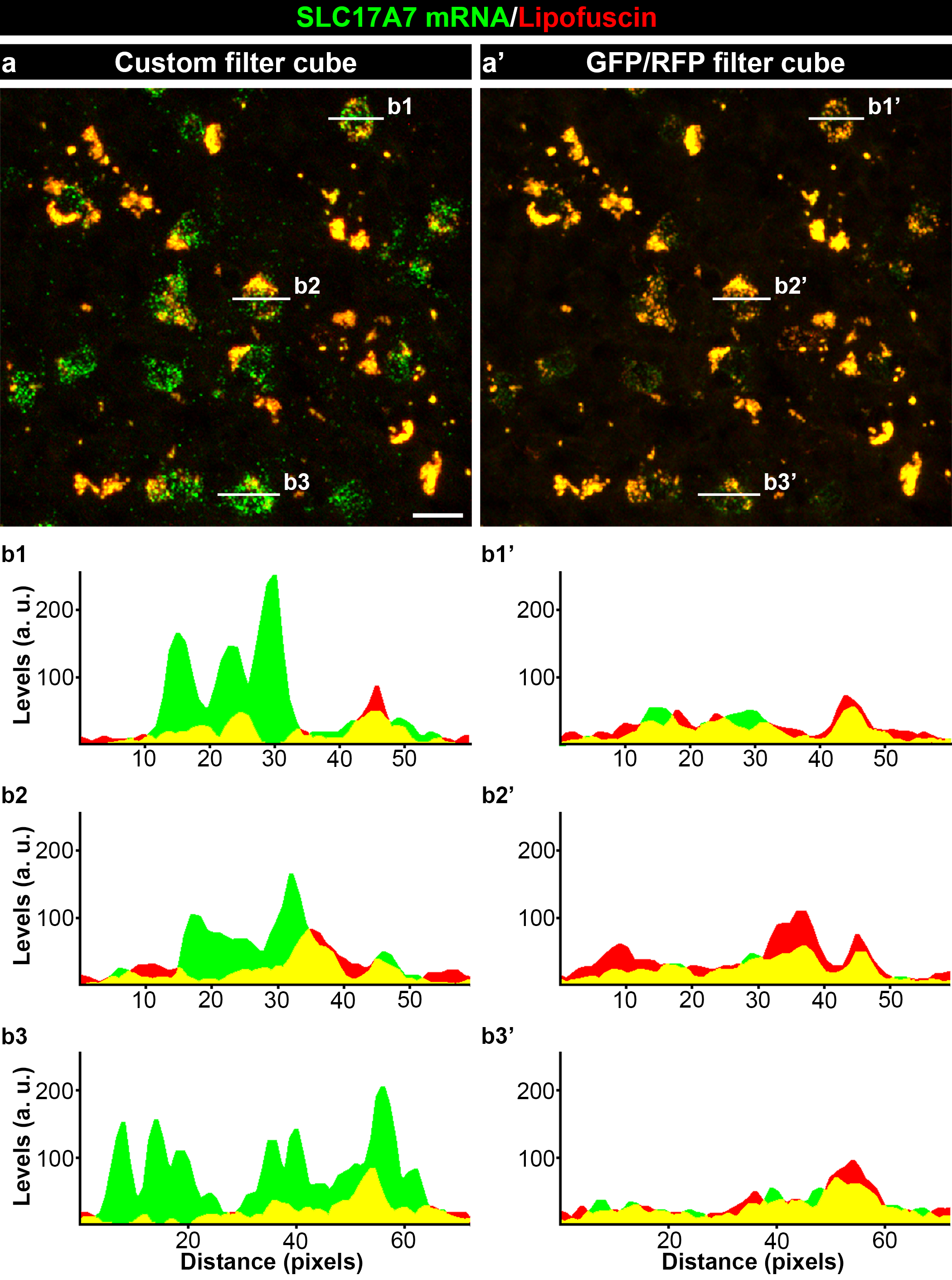
**

**Supplementary Figure S5.**

**
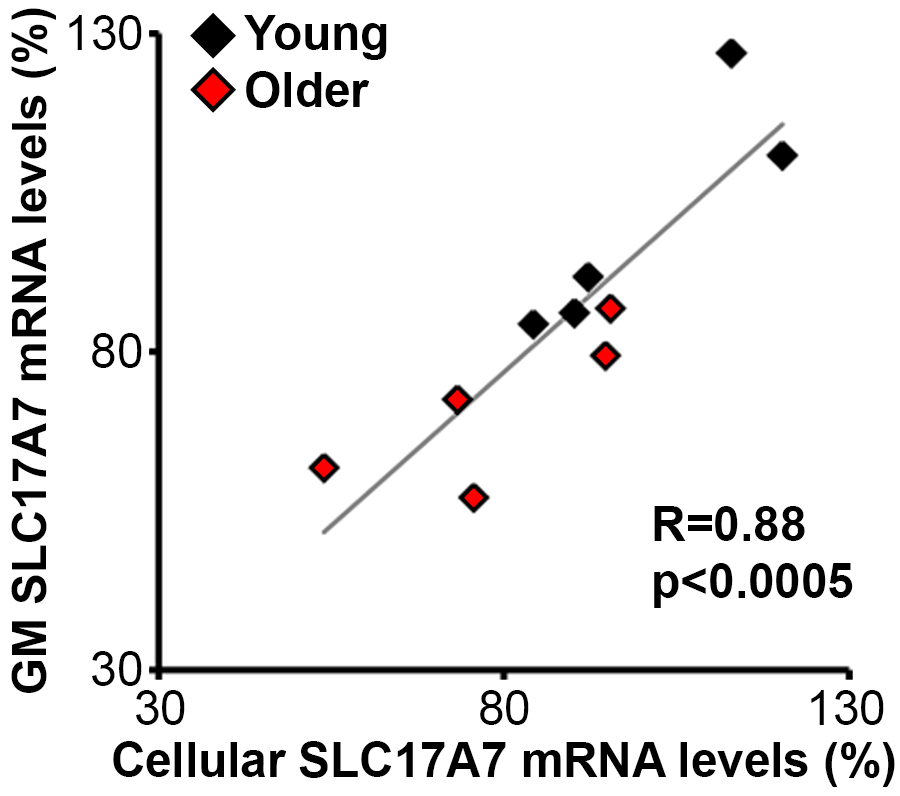
**

**Supplementary Figure S6.**

**
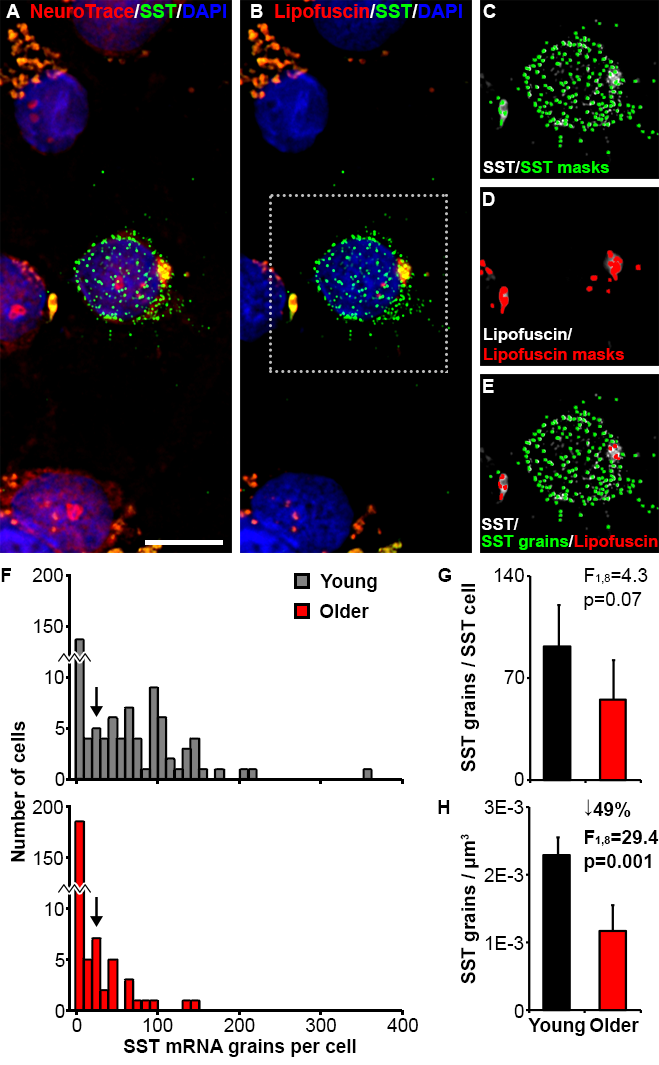
**

**Supplementary Figure S7.**


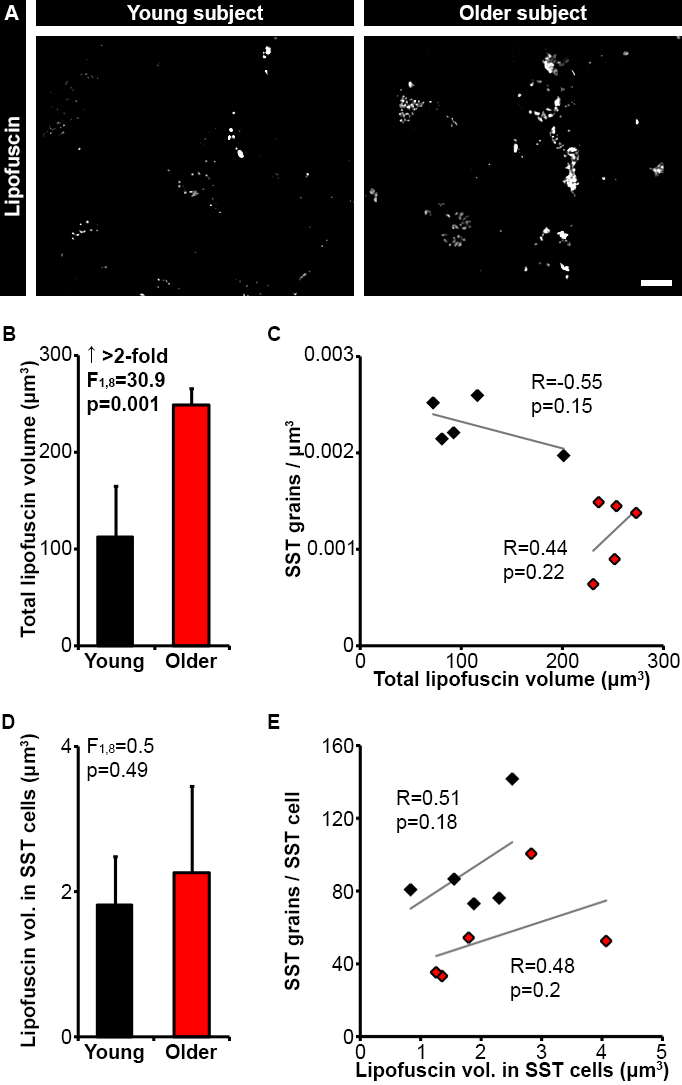


**Supplementary Figure S8.**


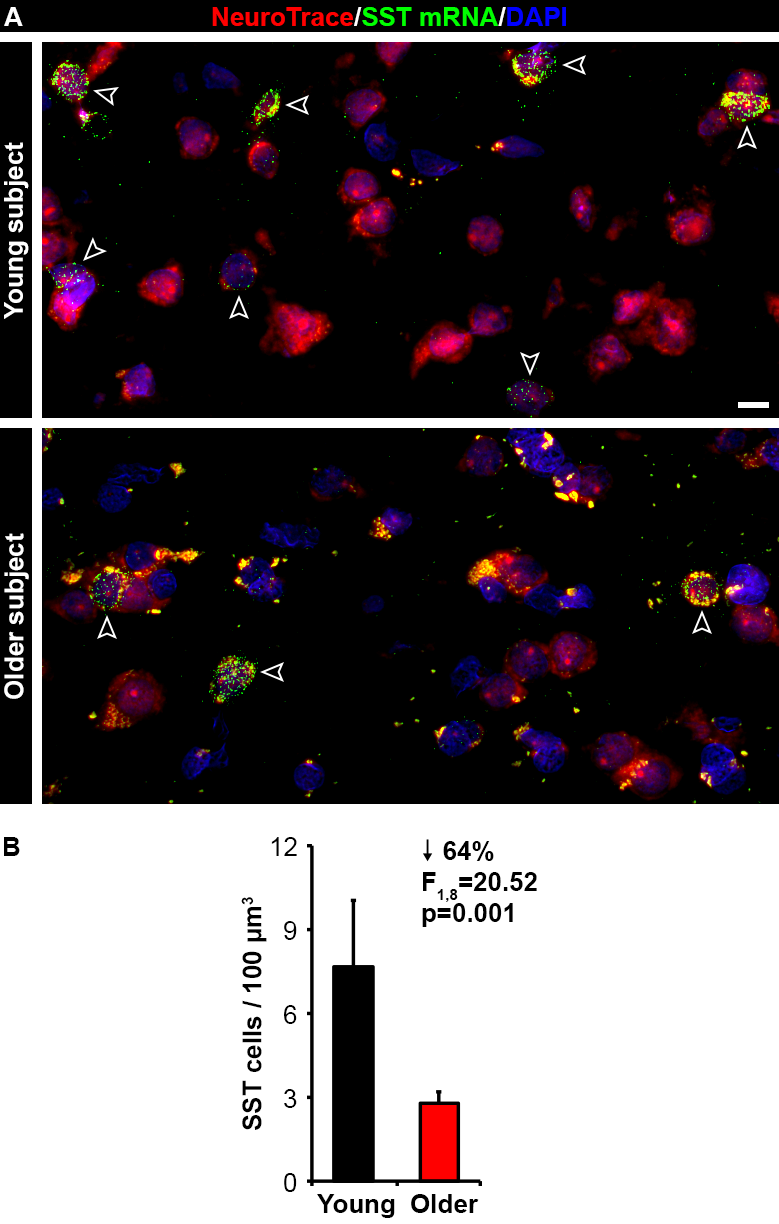


**Supplementary Figure S9.**

**
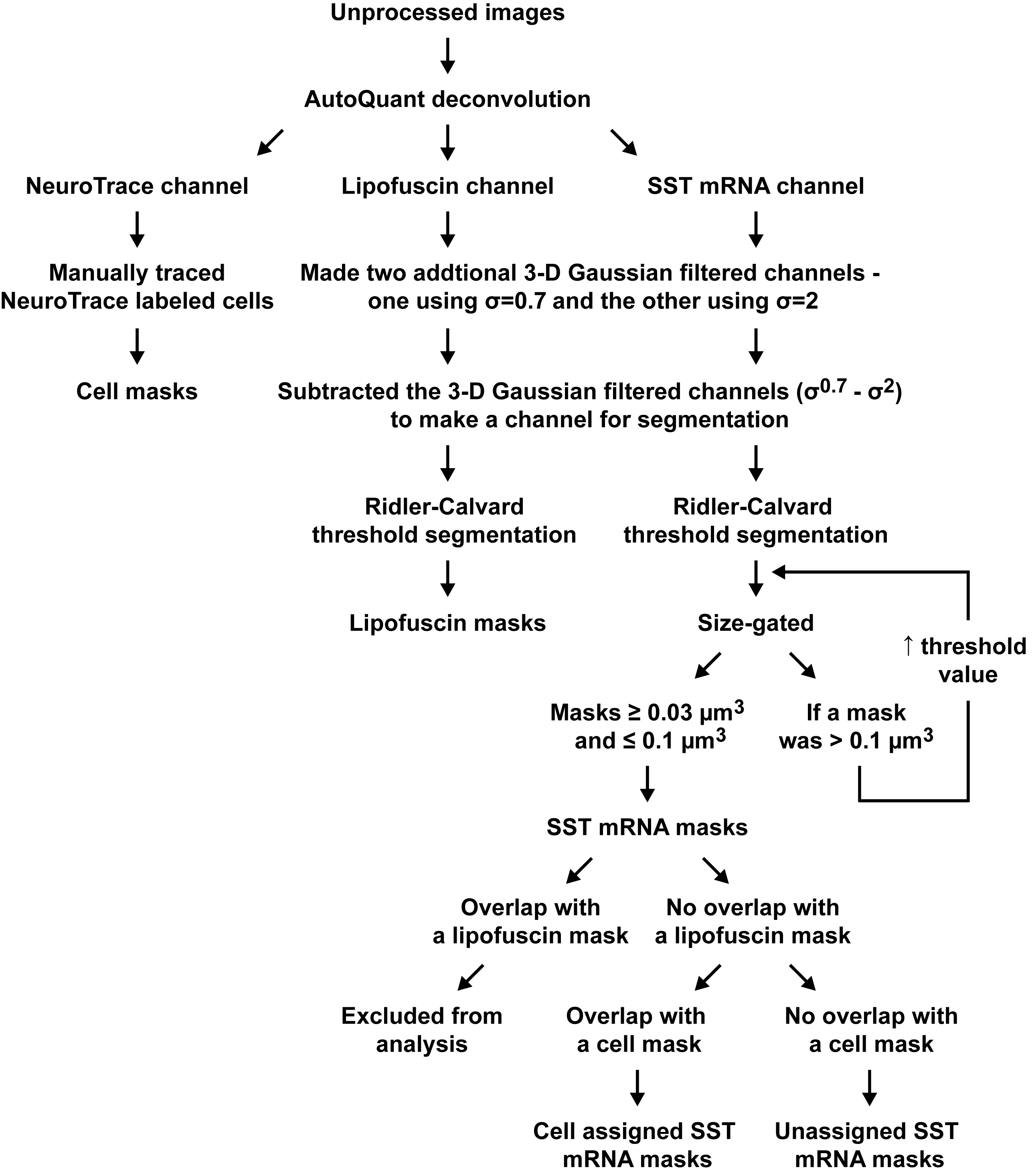
**

**SUPPLEMENTARY TABLES**

**Supplementary Table S1. Demographic and postmortem c**haracteristics of human subjects.

| **Young Subjects** | | | | | | | **Older Subjects** | | | | | | |
| --- | --- | --- | --- | --- | --- | --- | --- | --- | --- | --- | --- | --- | --- |
| **Case** | **Sex** | **Age (yrs)** | **PMI^A^** | **RIN^B^** | **pH** | **Cause of Death** | **Case** | **Sex** | **Age (yrs)** | **PMI^A^** | **RIN^B^** | **pH** | **Cause of Death** |
| 20 | M | 31 | 29.5 | 5.6 | 6.59 | Accident | 411 | M | 76 | 10.8 | 4.4 | 5.95 | Natural |
| 250 | M | 26 | 12.0 | 3.2 | 6.75 | Accident | 598 | M | 79 | 21.9 | 4.3 | 6.4 | Natural |
| 31 | M | 21 | 24.0 | 4.3 | 6.27 | Natural | 745 | M | 63 | 16.8 | 5.0 | 6.02 | Natural |
| 36 | M | 27 | 20.5 | 5.0 | 6.18 | Natural | 974 | M | 72 | 24.5 | 6.1 | 6.36 | Natural |
| 94 | M | 15 | 27.0 | 4.7 | 5.95 | Accident | 989 | M | 79 | 14.8 | 4.4 | 5.77 | Natural |
| Mean |  | 24.0 | 22.6 | 4.6 | 6.3 |  | Mean |  | 73.8 | 17.8 | 4.8 | 6.1 |  |
| s.d. |  | 6.2 | 6.8 | 0.9 | 0.3 |  | s.d. |  | 6.7 | 5.5 | 0.8 | 0.3 |  |

There was a significant difference between subjects groups for age (t(8)=12.2, p<0.0001). There were no group differences for PMI (t(8)=1.2, p=0.25), RIN (t(8)=0.5, p=0.61), or pH (t(8)=1.2, p=0.25).

^A^PMI, postmortem interval (hours)

^B^RIN, RNA integrity number

M, male

**Supplementary Table S2.** Relative expression levels in orbitofrontal cortex gray matter.

| **Young Subjects** | | | **Older Subjects** | | |
| --- | --- | --- | --- | --- | --- |
| **Case** | **SLC17A7 mRNA**  **levels (%)** | **SST mRNA**  **levels (%)** | **Case** | **SLC17A7 mRNA**  **levels (%)** | **SST mRNA**  **levels (%)** |
| 20 | 92.2 | 95.7 | 411 | 73.3 | 63.3 |
| 250 | 84.3 | 95.3 | 598 | 75.7 | 37.7 |
| 31 | 90.2 | 94.4 | 745 | 95.4 | 18.6 |
| 36 | 113.0 | 92.7 | 974 | 94.7 | 44.1 |
| 94 | 120.3 | 121.8 | 989 | 54.0 | 29.3 |
| Mean | 100.0 | 100.0 | Mean | 78.6 | 38.6 |
| s.d. | 15.7 | 12.3 | s.d. | 17.2 | 16.8 |

There was a trend towards statistical significance between groups for relative SLC17A7 mRNA expression levels (F(1, 8)=4.2, p=0.07) and a significant difference between subject groups for relative SST mRNA expression levels (F(1, 8)=43.6, p<0.0001) in gray matter homogenates of the orbitofrontal cortex.
